# Role of adipose-derived stem cells in healing surgically induced trauma of the rat’s tunica albuginea

**DOI:** 10.1093/sexmed/qfad058

**Published:** 2023-11-20

**Authors:** Abdallah Sharqawi, Mona F Mansour, Gamal A Elatrash, Ezzat A Ismail, David Ralph, Ahmed I El-Sakka

**Affiliations:** Department of Urology, Suez Canal University, Ismailia 4111, Egypt; Department of Physiology, Suez Canal University, Ismailia 4111, Egypt; Department of Urology, Suez Canal University, Ismailia 4111, Egypt; Department of Urology, Suez Canal University, Ismailia 4111, Egypt; Institute of Urology, University College of London Hospital, London W1G 8PH, United Kingdom; Department of Urology, Suez Canal University, Ismailia 4111, Egypt

**Keywords:** tunica albuginea, trauma, healing, stem cell

## Abstract

**Background:**

Injection of adipose-derived stem cells (ADSCs) into the injured tunica albuginea (TA) may prevent fibrosis, restore the balance between pro- and antifibrotic pathways, and potentially mitigate erectile dysfunction caused by abnormal TA healing.

**Aim:**

To assess the potential role of ADSC injection on structural, ultrastructural, functional, and molecular changes in surgically induced trauma of the rat’s TA.

**Methods:**

Forty adult male albino Wistar rats were divided into 5 groups of 8 rats each: group 1, sham; group 2, injury to TA without treatment; group 3, injury to TA and suture repair; group 4, injury to TA and injection of ADSCs without suture repair; group 5, injury to TA followed by injection of ADSCs and suture repair.

**Outcomes:**

After 6 weeks, all groups were subjected to functional, histologic, and ultrastructural examination and molecular expression of healing growth factors.

**Results:**

The intracavernous pressure (ICP; mean ± SD) was 114 ± 2, 32 ± 2, 65 ± 2, 68 ± 2, and 111 ± 2 mm Hg in groups 1 to 5, respectively. There were significant differences in ICP between each of groups 3 to 5 and group 2 (*P* < .05), and groups 3 and 4 each had significant differences with group 1 (*P* < .05). No significant difference in ICP occurred between groups 3 and 4 (*P* > .05). There were significant histologic and ultrastructural alterations in tunical tissues from group 2; however, these changes were markedly less in group 5 in terms of lower levels of fibrotic changes, elastosis, and superior overall neuroendothelial expression. Groups 3 and 4 showed improved structural and ultrastructural parameters when compared with group 2. Group 5 demonstrated lower levels of transforming growth factor β1 and basic fibroblast growth factor expression.

**Clinical Implications:**

This experimental model may encourage administration of ADSCs to prevent the deleterious effects of trauma to the TA.

**Strengths and Limitations:**

Injecting ADSCs can improve the healing process and erectile dysfunction in a rat model following TA injury, and combining ADSC injection with surgical suturing resulted in superior outcomes. The main limitation was the absence of long-term ICP measurements and a longer follow-up period that may provide further insight into the chronic phase of the healing process.

**Conclusion:**

ADSC injection may prevent structural, ultrastructural, functional, and molecular alterations in surgically induced trauma of the rat’s TA and enhance the effect of tunical suturing after trauma.

## Introduction

The tensile strength and elasticity of the tunica albuginea (TA) are crucial for achieving a full and rigid erection. Aberrant healing of the TA following repetitive minor sexual trauma, penile fractures, or certain penile surgery can lead to tunical fibrosis and Peyronie’s disease (PD).^1^^,^^2^ This abnormal healing process is characterized by a disorganized and altered ratio of elastin to collagen fibers, with fibrotic changes in the erectile tissue, which ultimately result in erectile dysfunction.^3^ The overexpression of profibrotic cytokines such as transforming growth factor β1 (TGF-β1), fibroblast growth factor (FGF), plasminogen activator inhibitor 1, and tissue inhibitors of matrix metalloproteinases is believed to be responsible for the pathophysiology of TA fibrosis and PD.^4^

Previous studies have shown that mesenchymal stem cells (MSCs) can differentiate into various mesenchymal lineages and are involved in wound healing through cellular differentiation, growth factors production, and immunomodulation.^5^ They also help in creating a conducive environment for the generation of other stem cells and proper signal transduction between epithelial and mesenchymal cells.^6^ By correcting the imbalance between pro- and antifibrotic pathways, MSC administration is thought to prevent fibrosis and positively affect immunosuppressive, immunomodulatory, and antioxidant pathways.^7-9^ Liposuction-obtained fat tissue is an excellent source of MSCs with low donor-site adverse events, and the extraction process is simple and produces a high MSC yield.^10^^,^^11^

Several animal studies have suggested that injecting adipose-derived stem cells (ADSCs) into the TA of a rat model with induced PD, during its acute or chronic phase, could be beneficial.^12-14^ However, there is a lack of information about the potential effects of ADSC injection on the structural, ultrastructural, functional, and molecular expressions of TGF-β1 and basic FGF in surgically induced trauma of the rat’s TA. The aim of our current study is to explore the role of ADSC injection in the healing process following trauma to the TA in a rat model.

The study hypothesis question was as follows: Does ADSC therapy induce improvement of functional, histologic, and ultrastructural aspects of surgically induced trauma of the rat’s TA?

## Methods

The current experimental study was conducted at the animal house and research laboratory at the physiology department of the Faculty of Medicine, Suez Canal University. Forty male albino Wistar rats aged 60 to 80 days and weighing 150 to 200 g each were sourced from the Cairo National Research Centre for experimental animals.

Environmental standardization for all animals was instituted per the National Research Council.^15^ To avoid bias, a unique identification number was given to each rat, which was randomly assigned to 1 of 5 groups (8 rats each).

*Group 1:* sham group—incision of the penile skin and the underlying fascia, then closure of skin and fascia without injury to the TA

*Group 2:* injury without treatment—incision of the TA, then closure of the fascia and skin without suturing of the tunica.

*Group 3:* suturing only—suture closure of the incised TA, then closure of the fascia and skin

*Group 4:* ADSC injection only—local injection of ADSCs into the edges of the incised TA, then closure of the fascia and skin without suturing of the TA

*Group 5:* ADSC injection and suturing—injections of ADSCs into the edges, then suturing of the incised TA followed by closure of the fascia and skin

### Surgical technique

**Figure 1:**
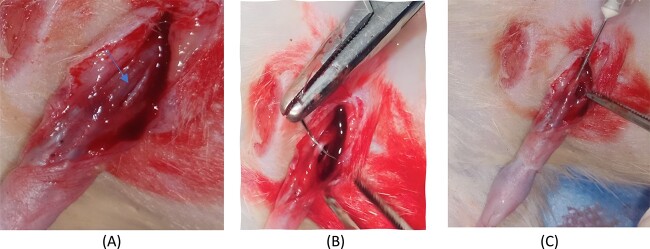
Tunica albuginea (A) tear (arrow), (B) suturing and (C) Adipose-derived stem cell injection.

An extended penile skin incision was made down to the pubic bone; then, a 1-cm incision was created in the dorsolateral aspect of the TA with a scalpel blade, which was subsequently closed with 6/0 Vicryl sutures under 5× magnification.^1^ To facilitate identification of the injured site later, a nonabsorbable suture was placed at the superior edge of the incised TA (Figure 1).

### ADSC collection and preparation

#### ADSCs were collected and prepared from the rat’s peritoneum (allogenic)

For rats receiving ADSC injection in groups 4 and 5, the process of ADSC separation included 2 major stages: isolation and digestion. Isolation of adipose tissue was collected from healthy rats following their scarification and mixed with an equivalent volume of a sterile phosphate-buffered saline containing 1%-5% penicillin/streptomycin/amphotericin. A clear adipose tissue was transferred to a sterile Petri dish after several steps of shaking and washing. For digestion with collagenase, approximately 2 mL of collagenase IV, prepared at a concentration of 1 to 2 mg/mL in TESCA buffer, was combined with minced adipose tissue. The stromal vascular fraction, which contains adipose stem cells, was isolated by centrifugation, plated into a sterile culture flask, and incubated at 37 °C and 5% CO_2_ for 72 hours.^16^

#### Flow cytometric assessment of cultured ADSCs

**Figure 2:**
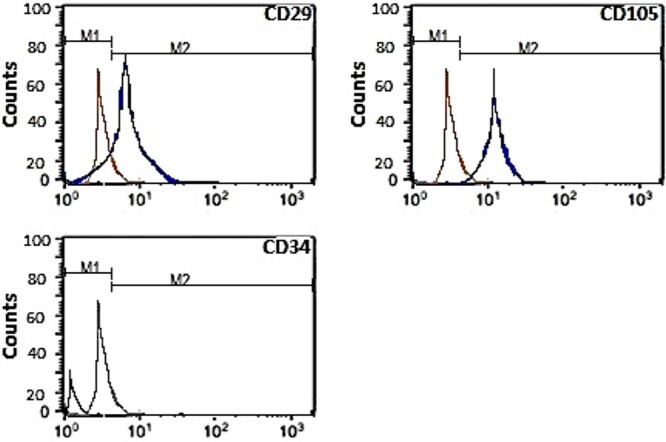
Flow cytometric assessment of cultured adipose-derived stem cells.

ADSCs were immunophenotypically characterized with CD29, CD105, and CD34 flow cytometry antibodies according to the manufacturer’s guidelines (Figure 2).^17^

#### Labeling of ADSCs

According to the manufacturer’s instructions, trypsinized ADSCs were labeled with a PKH26 fluorescent linker dye (MINI26; Sigma-Aldrich). A final concentration of 2 × 10^6^–M PKH26 dye and 1 × 10^6^ cells/mL was used in a total volume of 2 mL.^18^

#### ADSC transplantation

PKH26-labeled ADSCs were injected into 4 areas of injured TA edges on day 1 in a concentration of 1 million cells in 70 μL of phosphate-buffered saline.^19^

Six weeks later, all rats were subjected to functional evaluation of intracavernous pressure (ICP) before euthanasia and tissue harvesting. Tissue samples were prepared and examined for histologic, ultrastructural, and molecular assessments. Homing of transplanted ADSCs was done with a fluorescent microscope (Leistungselektronik Jena GmbH) in the TA of unstained random paraffin sections.

### Functional evaluation

The ICP was monitored and documented by the BIOPAC MP150 system connected to a computer device with AcqKnowledge software (version 4.1). To ensure accuracy, measurements were repeated several times. The NIBP200A noninvasive blood pressure system manufacturer’s guide (BIOPAC Systems Inc) was consulted to acquire recordings of the mean arterial pressure (MAP) via a tail-cuff monitor. For cavernous nerve electrostimulation, a steel bipolar hook electrode with 0.2-mm-diameter poles (1 mm apart) was used. Computer-generated monophasic squared pulses were delivered through a custom-constructed constant current amplifier, with electrical stimulus parameters as follows: 1.5-mA current, 20-Hz frequency, 0.2-mm pulse width, and 50-second duration.^20^

Following functional assessment, the marked site of the injured TA with the nonabsorbable suture was identified, and 2-mm segments of the TA and underlying cavernous tissue were taken for histopathologic, transmission electron microscopy (TEM), and molecular analysis. The tissue was placed in a 10% formaldehyde solution for light and fluorescent microscopy analysis. For TEM assessment, the isolated segments were maintained in a 2.5% glutaraldehyde solution and studied with a JEOL-2100 transmission electron microscope. Two corresponding sections were homogenized for analysis of FGF and TGF-β1 expression in the tissue.

### Determination of FGF and TGF-β1

The levels of FGF and TGF-β1 in the homogenized tissue were measured with enzyme-linked immunosorbent assays (ELISAs). A mouse/rat FGF basic/FGF2/bFGF immunoassay quantikine ELISA was used to measure FGF levels, while a human/mouse/rat/porcine/canine TGF-β1 immunoassay quantikine ELISA was used to measure TGF-β1 levels. The assays were performed according to the manufacturer’s instructions. Polyclonal microtiter wells were coated with antibodies, and neutralized standards and samples were added. Following this, a substrate solution was included, and the immunoenzyme sandwich formed. The color intensity that developed was in proportion with the concentration of TGF-β1/FGF detected in the examined tissue.^21^

### Histopathologic examination

Five-micron sections were stained with hematoxylin-eosin and Masson trichrome techniques to measure the thickness of the TA and check for inflammatory or fibrous cells. A digital microscope camera (UIS Optical System) was used to examine the samples, which featured a 10-megapixel resolution, 4356 × 2740 pixels per image, high-power field at 400×, and intermediate-power field at 100×.^22^

### Ultrastructural examination

TEM sections were assessed for ultrastructural changes of the TA (areas of smooth muscle fibers, elastic tissue, fibrous tissue, and microfibrils), vascular endothelial changes, and neuronal configurations.

### Statistical analysis

Data were collected and analyzed with SPSS software (version 20; IBM). Mann-Whitney *U* and Kruskal-Wallis tests were used to analyze differences in ICP, MAP, TGF-β1, and FGF expressions. *P* < .05 was considered statistically significant.

### Ethical considerations

The study followed the National Research Council’s guidelines for animal experimentation, ensuring that the animals did not experience unnecessary suffering and were humanely euthanized at the end of the study. Veterinarians monitored the animals’ welfare and ensured their access to proper nutrition. The study was approved by the Ethics Committee of the Faculty of Medicine, Suez Canal University (research 3599). Furthermore, we took diligent care to adhere to the guidelines outlined in the ARRIVE checklist (Animal Research: Reporting of In Vivo Experiments) throughout the entirety of our study’s design.

## Results

### Functional assessment: ICP and MAP measurements

**Figure 3:**
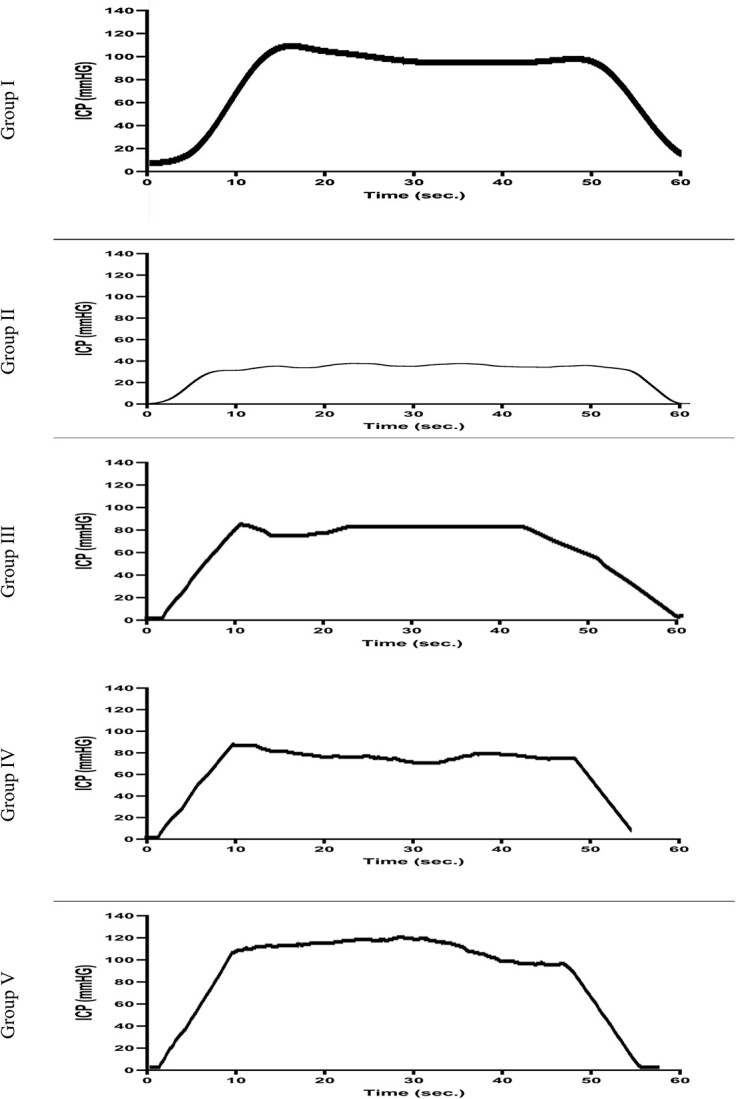
Comparison of ICP measurements among study groups. In the context of group 1, upon cavernous nerve stimulation, the ICP curve exhibited successful attainment and sustained maintenance of an erection throughout the entire period of stimulation. In contrast, the ICP curve for group 2 depicted suboptimal parameters, reflecting a less favorable erectile response. For groups 3 and 4, the ICP parameters exhibited reasonable values; however, a significant duration of time elapsed after the stimulation before the attainment of a full erection vs group 1. Group 5 displayed an ICP curve that closely paralleled the pattern observed in group 1. ICP, intracavernous pressure.

**Figure 4:**
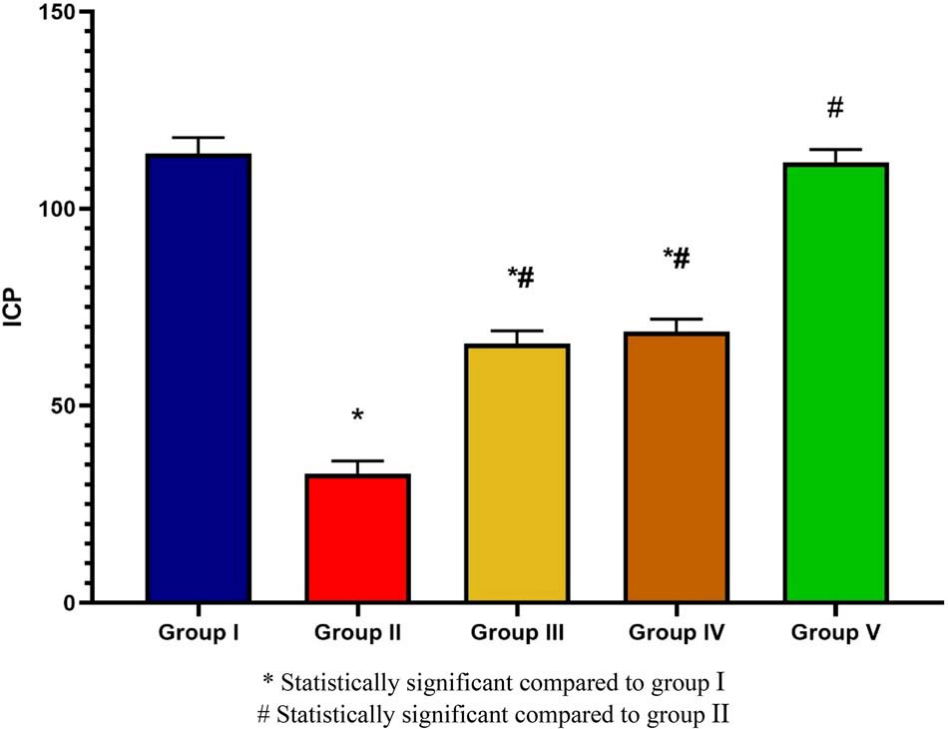
Changes in ICP among study groups. There were significant differences in ICP between each of groups 3, 4, and 5 and group 2; likewise, each of groups 3 and 4 had a significant difference with group 1. There was no significant difference in ICP between groups 3 and 4. ICP, intracavernous pressure.

The ICP for groups 1 to 5 was 114 ± 2, 32 ± 2, 65 ± 2, 68 ± 2, and 111 ± 2 mm Hg, respectively. When individual comparisons were carried out, as expected, groups 3 and 4 had a significantly lower ICP than group 1 (*P* < .05). However, we noted no difference in ICP between group 5 and the sham group (*P* > .05). The ICP in the suturing-only and ADSC injection–only groups was also not significant (*P* > .05). The group that underwent injury without treatment had a significantly lower ICP vs all the other groups (group 1, *P* = .001; group 3, *P* = .02; group 4, *P* = .011; group 5, *P* = .001). There was no significant difference in MAP among the study groups (*P* = .75; Table 1, Figures 3 and 4).

**Table 1:** Differences in MAP.

Group^a^	MAP, mm Hg, mean ± SD
1: Sham	111 ± 4
2: Injury without treatment	106 ± 2
3: Suturing only	110 ± 2
4: ADSC injection only	108 ± 5
5: ADSC injection and suturing	110 ± 5

Abbreviations: ADSC, adipose-derived stem cell; MAP, mean arterial pressure.

a All groups: *P* = .753 (Kruskal-Wallis test).

### Histopathology

**Figure 5:**
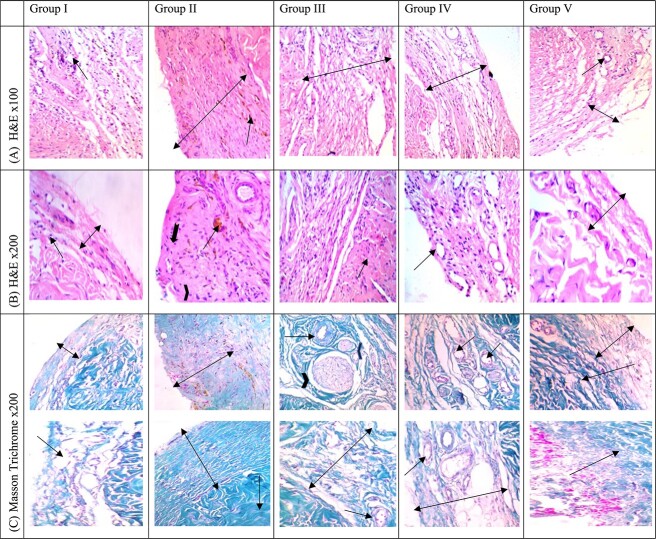
Comparison of (A, B) hematoxylin-eosin and (C) Masson trichrome histopathologic sections among study groups. Group 1: Examined sections revealed portions of TA and corpora cavernosa with luminal vascular spaces lined by endothelial cells surrounded by smooth muscle bundles (long arrows), covered by a thin layer of TA formed of thin collagen bundles (double arrows), and no evidence of fibrosis with trichrome staining. Group 2: The TA was markedly thickened (double arrows), appearing as thick collagen bundles surrounding corporeal tissue with severe adhesions, marked increased cellularity formed of proliferating fibroblasts (spindle shaped cells = chevrons), inflammatory cells (rounded cells lymphocytes = notched arrows), hemosiderin-laden macrophages (long arrows) with proliferating vessels, and a noticeable increase in fibrous tissue (long arrows) with Masson trichrome stain. Groups 3 and 4: The TA was less thickened vs group 2 (double arrow), with increased cellularity of proliferating fibroblasts (long arrows), no inflammatory cell infiltration, a limited number of proliferating vessels (chevrons), mild edema, and increased collagen deposition with less space within the cavernosa (short arrow). Masson stain showed deep staining and increased fibrosis (double arrows); entrapments of few nerves were also noticed (chevrons). Proliferating vessels were present; however, they were encircled by prominent adhesions (long arrows). Group 5: Thin TA formed of thin collagen bundles (double arrows) with decreased cellularity, few fibroblasts, and a limited number of vessels (long arrows), which denote a well-settled healing process; neither inflammatory cells nor adhesions (short arrows) were present. Masson stains highlight less fibrosis and preserved cavernous spaces (long arrows). TA, tunica albuginea.

After comparing hematoxylin-eosin and Mason trichrome histopathologic sections among study groups, we found that group 5 had minimal alterations in the cellular architecture of the TA and cavernous tissue, with an overall appearance similar to group 1. This was characterized by a thin layer of TA formed of collagen bundles, and there was no evidence of inflammatory cells, adhesion, or fibrosis. Yet, group 2 showed a distorted architecture of the TA and underlying cavernous tissues, with marked adhesions and increased cellularity, as well as the presence of proliferating fibroblasts, inflammatory cells, and hemosiderin-laden macrophages. Groups 3 and 4 exhibited less preserved architecture of the TA and cavernous tissue when compared with groups 1 and 5. Mild adhesions and increased cellularity, including fibroblasts and inflammatory cells, were observed in these groups. Proliferating vessels were present, indicating an attempted tissue repair response (Figure 5).

### Detection of ADSC homing in the TA

**Figure 6:**
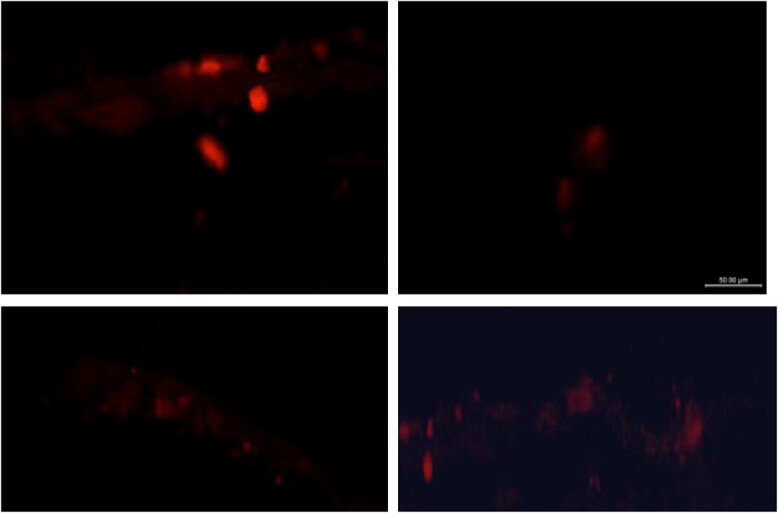
Homing detection of the injected PKH26 dye fluorescent–labeled adipose-derived stem cells in the tunica with fluorescent microscopy.

Fluorescent microscopic examination of the TA tissue showed intense staining of PKH26 fluorescent-labeled ADSCs within the groups that received ADSC injection treatment (groups 4 and 5; Figure 6), which confirms homing and survival of the injected ADSCs in the TA. Fluorescent emissions were absent in other groups without ADSC treatment (groups 1-3).

### Ultrastructural assessment

**Figure 7:**
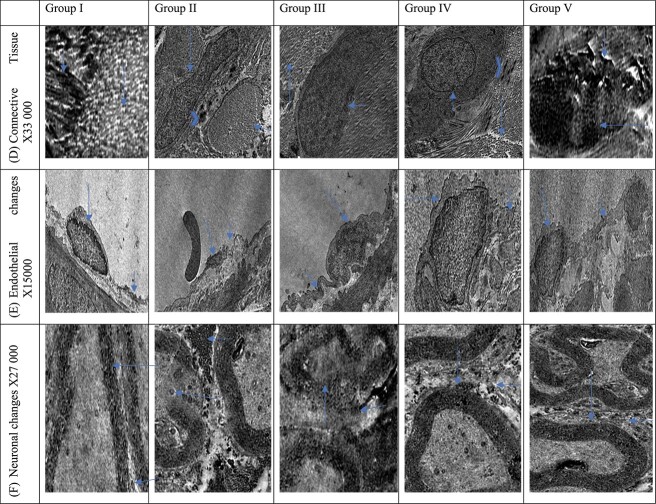
Transmission electron microscopy sections show changes in connective tissue and endothelial and neuronal ultrastructural among study groups. Groups 1 and 5: Transmission electron microscopy section illustrates normally distributed organized collagen fibers (long arrow) with islands of normal elastic fibers (short arrow), a regularly preserved capillary basement membrane (long arrow), preserved endothelial nuclear chromatin (euchromatic; short arrow), and intact nucleolus and cytoplasm, with less collagen fiber deposition around the capillaries, a maintained structure of myelinated nerve fibers, and an intact myelin sheath (long arrows), as well as preserved perineural space and no collagen fiber deposition in the perineural space (short arrow). Group 2: A heterochromatin nucleus of fibroblast was noted (long arrow) with packed collagen fiber bundles with loss of collagen bundle sheath (short arrow) and few fragmented elastic fibers (chevron), a distorted capillary basement membrane (long arrow), shrunken atrophic endothelial nuclei (short arrow), and a disfigured nuclear membrane, as well as distorted Schwann sheath myelination (long arrow) and increased perineural space; moreover, numerous collagen fibers were identified in a remarkably widened perineural space (short arrows). Groups 3 and 4: The ultrastructural alterations were nearly comparable and demonstrated moderately dense collagen fibers (long arrow), regular contour fibroblast cells, and preserved nuclear chromatin (short arrow), but few elastic fibers were present (chevron); an intact but irregular capillary basement membrane (long arrow) was identified with a euchromatic endothelial nucleus and preserved nucleolus and cytoplasm, as well as moderately dense collagen fibers around capillaries (short arrow), increased perineural space, mild collagen fiber deposition in the perineural space (short arrow), and preserved but less myelinated nerve fibers (long arrow).

In groups 1 and 5, TEM sections revealed normally distributed organized collagen fibers with preserved endothelial ultrastructure and intact myelin sheath in nerve fibers. Group 2 had numerous packed collagen bundles with distorted capillary basement membrane, loss of Schwann sheath myelination, and numerous collagen fibers in the perineural space. The ultrastructural alterations were significantly marked in groups 3 and 4 vs groups 1 and 5 but less pronounced than in group 2. TEM sections in groups 3 and 4 showed moderately dense collagen fibers around capillaries, increased perineural space, and mild collagen fiber deposition in the perineural space, with preserved myelin sheath (Figure 7).

### Determination of TGF-β1 and FGF

**Figure 8:**
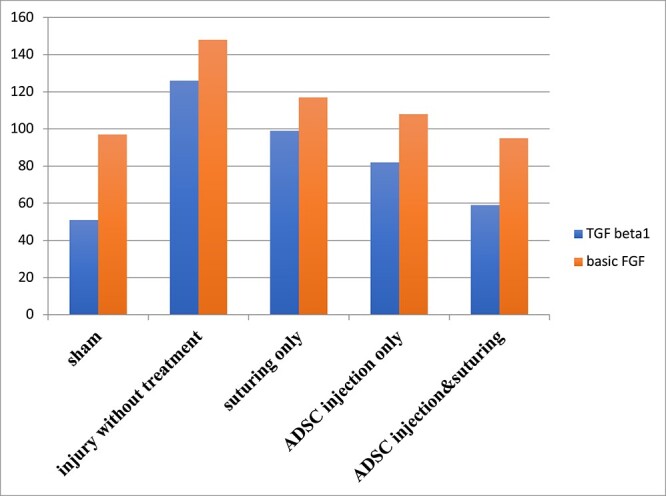
The difference in TGF-β1 and FGF among the study groups. Enzyme-linked immunosorbent assay levels of TGF-β1 were 51 ± 2, 126 ± 2, 99 ± 2, 82 ± 2, and 59 ± 2 pg/mg of protein in groups 1 to 5, respectively, while basic FGF levels were 97 ± 4, 148 ± 2, 117 ± 2, 108 ± 5, and 95 ± 5 pg/mg of protein. FGF, fibroblast growth factor; TGF-β1, transforming growth factor β1.

There was significant upregulation of TGF-β1 and basic FGF in group 2 when compared with each of groups 1, 3, 4, and 5. A statistically significant difference in TGF-β1 and basic FGF levels was noticed with upregulation in groups 3 and 4 when compared with groups 1 and 5 (*P* < .05) each. There was no significant difference in TGF-β1 and basic FGF levels between groups 5 and 1 (*P* > .05); similarly, no significant difference was observed between groups 3 and 4 (*P* > .05; Figure 8).

## Discussion

Stem cell therapies represent a burgeoning and prospective domain of research that offers the potential to heal diseases related to fibrotic conditions; specifically, they can mitigate fibrosis by taking part in manifold antifibrotic stages.^23-26^

In an experimental model, the current study demonstrated that combining TA suturing and ADSC injection after TA injury has superior healing benefits. This combined treatment resulted in reduced fibrotic changes and elastosis, maintained neuroendothelial appearances, and caused lower levels of TGF-β1 and FGF expression. The outcome was reflected as an improved ICP equivalent to an uninjured TA. Single-treatment modalities showed comparable improvements in overall parameters but were inferior to the combined treatment. 

In this study, ADSCs labeled with fluorescent PKH26 dye were observed to accumulate at the site of injury in the TA at levels that suggest adequate homing and survival, which is consistent with findings from previous studies with similar methods.^13^^,^^18^ This increased signal intensity and homing of ADSCs at the site of injury support the idea that the TA is receptive to ADSCs and that they may have a therapeutic effect.

High levels of fibrotic cytokines, such as TGF-β1 and basic FGF, are associated with excessive fibrosis in healing tissues. TGF-β1 upregulation has been confirmed in PD and after TA injury.^27-29^ Similarly, previous research found a significant increase in basic FGF expression in PD. Other studies have highlighted the important role of basic FGF in fibrosis and renal scarring.^30-32^ Nevertheless, this study demonstrated that when TA injury was treated with a combination of suture repair and ADSC injection, there were notably lower levels of TGF-β1 and basic FGF than in other study groups. These reduced levels of fibrotic factors suggest that there was less fibrosis surrounding the injury site.

The functional assessment of the study groups showed no significant difference in ICP levels between the suturing- and ADSC injection–only groups. However, the combined treatment of suturing and ADSC injection resulted in a better outcome. These findings are consistent with previous studies that reported functional improvement in erectile function following ADSC injection in rat models with induced PD.^13^^,^^33^^,^^34^

The histopathologic examination in the current study revealed significant differences in the TA’s thickness and collagen bundle architecture and the presence of inflammatory cells and fibroblasts among the study groups. The combined treatment group showed minimal alteration in cellular architecture of the TA and cavernous tissue and an overall tissue appearance nearly similar to the sham group. The group that underwent injury without treatment showed gross distortion of architecture in the TA and underlying cavernous tissues, with marked thickening of the TA, widespread adhesions, and heavy cellular infiltration. The overall histologic appearance in the suturing-only and ADSC injection–only groups was better than the group with injury without treatment but less favorable than the combined treatment group. El-Sakka et al found that tunical injury led to structural changes and a PD-like condition with the release of TGF-β1 and infiltration of inflammatory cells.^35^ Castiglione et al observed excessive fibrosis and elastosis in the PD group, while the ADSC injection group had better-organized collagen fibers and sinusoidal structures.^12^ Several studies have reported similar histologic changes, including increased tunica thickness, disorganized trabeculae, and varying degrees of fibrosis in the PD group and better-organized trabeculae, average tunica, and minimal fibrosis in the ADSC-treated groups.^34^^,^^36^^,^^37^

A prior study examined the ultrastructural changes linked to PD and found an increase in collagen bundle density and perineural and perivascular changes.^35^ These results are consistent with our findings, which were particularly noticeable in the untreated injury group. However, in the group that received combined treatment, the nuclear membrane, perineural space, and nuclear integrity remained intact, with an undamaged basal lamina of the endothelial capillaries and minimal collagen fiber deposition. Varying levels of changes in connective tissue and neuroendothelial appearance were observed in the groups that received only suturing or ADSC injection, but the overall ultrastructure was not as severely altered as in the untreated injury group.

Although the end point assessment in the current study was carried out at 6 weeks after TA injury to ensure a settled healing process, further study with longer follow-up assessment may add merit in understanding the chronic phase of the healing process. Another limitation of this study is the absence of long-term ICP measurements, which could not confirm whether the functional improvement observed following ADSC treatment is sustained or temporary. Nevertheless, the current study has demonstrated that injecting ADSCs after TA injury can alleviate the negative consequences of abnormal TA healing and subsequent erectile dysfunction in a rat model. It was observed that ADSC injection had similar efficacy to surgical suturing of the TA; as such, these findings suggest that ADSC treatment shows promise as a potential bedside therapy for penile trauma. Nonetheless, additional research, including rigorous preclinical and clinical trials, is necessary to comprehensively validate its efficacy and ascertain its feasibility as a viable treatment option. Moreover, combining ADSC injection and surgical suturing after TA injury showed superior healing and functional outcomes that were almost equivalent to those of an uninjured TA.

## Conclusion

ADSC injection may prevent structural, ultrastructural, functional, and molecular alterations in surgically induced trauma of the rat’s TA. These results could be of significant clinical relevance, as administration of ADSCs may be a viable approach to reducing the adverse effects of TA trauma and maintaining erectile function in cases of penile trauma, fractured penis, and PD, as well as improving the outcomes of penile surgery.

## Data Availability

The data sets generated and/or analyzed during the current study are not publicly available, but they are available from the corresponding author on reasonable request.
